# Exploring the Effects and Mechanisms of Valerian Volatile Oil in Treating Insomnia Using Network Pharmacology, Molecular Docking, and Molecular Dynamics Simulation-Based Approaches

**DOI:** 10.3390/ijms26041726

**Published:** 2025-02-18

**Authors:** Halimulati Muhetaer, Huajian Li, Bingna Wang, Xinyi Cai, Yang Zhang, Yongxian Li, Chuwen Li, Bo Wu

**Affiliations:** Guangzhou Municipal and Guangdong Provincial Key Laboratory of Molecular Target & Clinical Pharmacology, The NMPA and State Key Laboratory of Respiratory Disease, School of Pharmaceutical Sciences, Guangzhou Medical University, Guangzhou 511436, China; hali0322001x@163.com (H.M.); 2022211570@stu.gzhmu.edu.cn (H.L.); 2023211667@stu.gzhmu.edu.cn (B.W.); 2023211636@stu.gzhmu.edu.cn (X.C.); 2024211824@stu.gzhmu.edu.cn (Y.Z.); 15088025425@163.com (Y.L.)

**Keywords:** valerian volatile oil, insomnia, network pharmacology, molecular dynamics, monoamine oxidases

## Abstract

Valerian possesses a multitude of pharmacological effects, including sedative and hypnotic properties, antihypertensive effects, antibacterial activity, and liver protection. Insomnia, one of the most prevalent disorders in contemporary society, significantly impacts people’s daily lives. This study aims to explore the anti-insomnia effects of valerian volatile oil (VVO) and investigate its potential mechanism of action through chemical analysis, network pharmacology, molecular docking, molecular dynamics simulations, and experimental validation. Through gas chromatography–mass spectrometry (GC-MS) analysis and drug-likeness screening, we identified 38 active compounds. Network pharmacology studies revealed that these 38 compounds might affect 103 targets associated with insomnia, such as monoamine oxidase B (MAOB), dopamine receptor D2 (DRD2), monoamine oxidase A (MAOA), interleukin 1β (IL1B), solute carrier family 6 member 4 (SLC6A4), prostaglandin-endoperoxide synthase 2 (PTGS2), and 5-hydroxytryptamine receptor 2A (HTR2A), which contribute to regulating the neuroactive ligand–receptor interaction, 5-hydroxytryptaminergic synapse, and calcium signaling pathways. The results of the molecular dynamics simulations indicated that bis[(6,6-dimethyl-3-bicyclo[3.1.1]hept-2-enyl)methyl] (E)-but-2-enedioate exhibited a stabilizing interaction with MAOB. The animal studies demonstrated that gavage administration of a high dose (100 mg/kg) of VVO significantly diminished autonomous activity, decreased sleep latency, and extended sleep duration in mice. Furthermore, the results of the Western blot experiment indicated that VVO interacts with MAOB, resulting in decreased expression levels of MAOB in the cerebral cortex. This study demonstrates the protective mechanism of VVO against insomnia through chemical analysis, network pharmacology, and experimental validation and extends the possible applications of VVO, which is a potential therapeutic ingredient for use in insomnia treatment.

## 1. Introduction

Insomnia is the most prevalent sleep disorder and constitutes a significant health problem that impacts the majority of individuals. This disease is primarily defined by difficulty initiating or sustaining sleep and the inability to resume sleep after awakening in the morning [1,2]. This condition is typically more common among older people than the younger population [3]. Still, according to a previous study, the incidence of insomnia is gradually showing a trend toward affecting younger people [4]. In addition, sleeplessness results in physical and psychological health issues, affecting patient’s daily work and overall lives. Research indicates that chronic insomnia is a considerable risk factor for cardiovascular disease, hypertension, type II diabetes, gastric reflux, and asthma [5,6,7,8,9,10]. Additionally, it can impair the health-related quality of life of patients with multiple sclerosis [11]. Therefore, insomnia has long been an important issue affecting public health, creating a massive market for the research and development of sleep-improving drugs.

Currently, pharmacological interventions for insomnia mainly consist of chemical drugs, broadly categorized into benzodiazepine receptor agonists, melatonin receptor agonists, histamine antagonists, etc. [12,13,14,15]. Prolonged usage of these substances is typically required to address persistent insomnia. However, extended consumption can lead to drug dependency, tolerance, addiction, and side effects, including amnesia, along with other harmful effects [16,17,18]. Alternatively, studies have shown that ingredients in natural herbs can have a soothing and hypnotic effect. Therefore, investigations into natural medications that are highly effective, non-toxic, and safe for prolonged use have emerged as a novel approach in the pharmaceutical management of insomnia. The aqueous extract from Flos Albiziae and its constituent quercetin can synergize with 5-hydroxytryptophan and antagonize p-chlorophenylalanine, shortening sleep latency and prolonging sleep duration, which may be related to the serotonergic system [19]. Schisandrin B, an active lignan component in Schisandra chinensis Turcz, reduces the mobility of mice and exerts a sedative–hypnotic effect by up-regulating the expression of GABAA receptors and regulating the levels of GABA and Glu in peripheral blood and brain tissue [20]. It has also been found that inhaling perilla essential oil also has sedative and hypnotic effects [21]. Therefore, traditional Chinese medicines and their active ingredients hold great potential in the treatment of insomnia and deserve further research.

*Valeriana officinalis* L. is a perennial herb rich in lignans, iridoids, flavonoids, and volatile oils. Its role as a traditional herb is essential in maintaining human health. Modern pharmacological studies have shown anticancer [22], anti-anxiety and depression [23,24], neuroprotective [25], and anti-epileptic [26] effects, and it is commonly used to treat insomnia. Several studies have indicated that the combination of *Eschscholtzia californica* Cham and *Valeriana officinalis* L. extracts enhances sleep efficiency and diminishes the frequency of awakenings; however, it does not affect sleep latency [27]. In addition, using valerian and lemon balm may assist women suffering from sleep disturbances or problems achieving sufficient sleep quality throughout menopause [28]. Choi et al. found that valerian, cascade, and valerian/cascade mixtures significantly reduced sleep latency and increased non-rapid eye movement sleep and total sleep time. They demonstrated that the mechanism of action was due to the upregulation of GABAA receptors [29]. Due to the variety of active compounds in valerian, while some studies have demonstrated its efficacy in producing sedative–hypnotic effects, there is a lack of research regarding its anti-insomnia active ingredients and mechanisms of action. Consequently, this study utilized network pharmacology to initially identify the sedative–hypnotic pharmacodynamic compounds, associated targets of probable effects, and prospective action routes, thereby offering a theoretical foundation for subsequent experimental investigations.

## 2. Results

### 2.1. GC-MS Analysis

**Figure 1:**
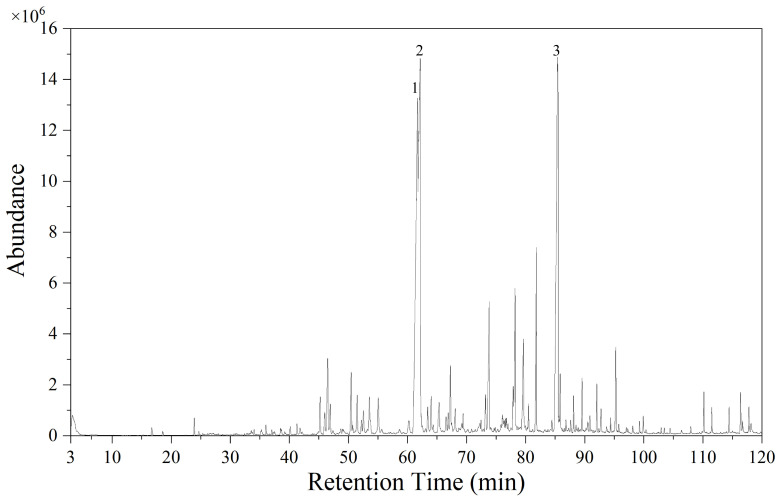
Total ion flow diagram of valerian volatile oil. 1:calarene; 2:ylangenal; 3:4-(3-methyl-1H-pyrazol-1-yl) aniline.

GC-MS was used to analyze the composition of VVO, and its total ion chromatogram was obtained (Figure 1). The NIST20 mass spectrometry database was searched and compared with select compounds with a match greater than or equal to 50. The relative percentage content of each chemical constituent was then calculated using peak area normalization.

In valerian volatile oil, 84 components were identified, accounting for 80.97% of the total volatile oil composition. Among them, there were three components had high contents, accounting for 50.63% of the total composition of the volatile oil, including 17.52% calarene (1), 16.83% ylangenal (2), and 16.28% 4-(3-methyl-1H-pyrazol-1-yl) aniline (3).

### 2.2. Target Screening of VVO

The 84 identified ingredients were screened through the SwissADME platform, and 38 active ingredients were obtained, as shown in Table 1. A total of 1373 potential targets of these active ingredients were predicted using the SwissTargetPrediction database. After duplicated genes were deleted, a final total of 418 potential targets of active ingredients were obtained.

### 2.3. Prediction of Insomnia Targets and Screening of Intersecting Targets

**Figure 2:**
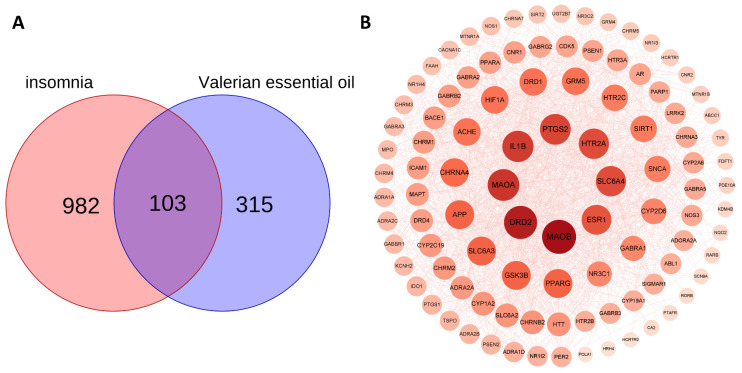
(**A**) Venn diagram of VVO constituent targets and disease targets. (**B**) PPI network of intersecting targets.

Insomnia targets were searched using the GeneCards, DrugBank, and TTD databases, and the collected results were de-duplicated. In total, 1085 disease targets were collected. Afterward, the screened active ingredient targets and disease targets were imported into the BioLadder (https://www.bioladder.cn/, accessed on 5 March 2024) platform to identify the intersection (Figure 2A), and 103 common targets were obtained as potential targets of valerian volatile oil for the treatment of insomnia.

### 2.4. Construction of a PPI Network

A total of 103 anti-insomnia targets of VVO were imported into the STRING platform to create a PPI network, and targets not involved in protein interactions were eliminated. The constructed network consisted of 102 nodes and 753 edges, and the average degree of the nodes was 14.8. The nodes’ sizes and colors were set according to their degree values, and a concentric circle was drawn. The higher the degree value, the larger and darker the node, which is located at the center of the concentric circles, as shown in Figure 2B. Among them, monoamine oxidase B (MAOB, degree = 82), dopamine receptor D2 (DRD2, degree = 78), monoamine oxidase A (MAOA, degree = 72), interleukin 1 beta (IL1B, degree = 70), prostaglandin-endoperoxide synthase 2 (PTGS2, degree = 68), 5-hydroxytryptamine receptor 2A (HTR2A, degree = 66), solute carrier family 6 member 4 (SLC6A4, degree = 66), and estrogen receptor 1 (ESR1, degree = 64) were the top eight targets in terms of their degree values.

### 2.5. GO Functional and KEGG Pathway Enrichment Analysis

**Figure 3:**
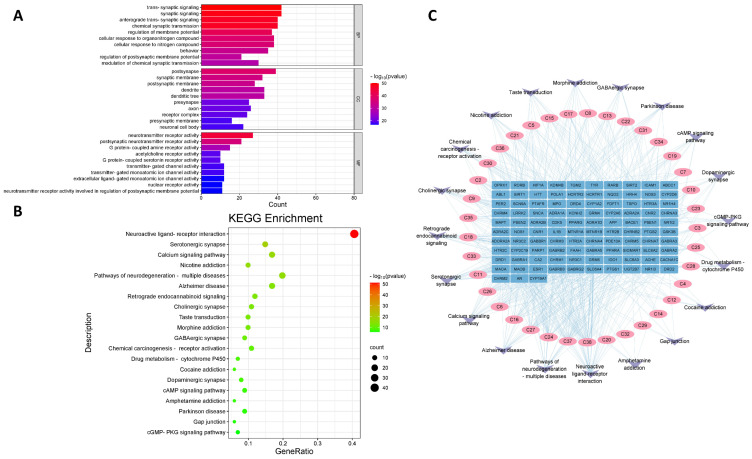
(**A**) GO functional enrichment analysis. (**B**) KEGG pathway enrichment analysis. (**C**) “Component-target-pathway” network.

We performed GO functional enrichment analysis to elucidate the molecular mechanism of action of the sedative–hypnotic effect of VVO. GO enrichment analysis using the Metascape database yielded 1223 entries (*p* < 0.01), which included 1005 entries for biological processes (BP), 77 entries for cellular components (CC), and 141 entries for molecular functions (MF). The top 10 ranked entries were selected according to the size of the *p*-value to plot the GO functional analysis (Figure 3A). The results show that the targets were mainly enriched in trans-synaptic signaling, synaptic signaling, anterograde trans-synaptic signaling, and other biological processes. Their main molecular functions were associated with neurotransmitter receptor activity, postsynaptic neurotransmitter receptor activity, and G protein-coupled amine receptor activity. The cellular components are mainly involved in the postsynaptic, synaptic, and postsynaptic membranes.

By using the Metascape (https://metascape.org/, accessed on 15 March 2024) database, 55 KEGG signaling pathways were obtained (*p* < 0.01). The top 20 pathways were selected according to the size of the *p*-value to plot the KEGG enrichment analysis results, as shown in Figure 3B. The KEGG results show that the targets were mainly enriched in the following pathways: the neuroactive ligand–receptor interaction, serotonergic synapse, calcium signaling pathway, nicotine addiction, and pathways related to neurodegeneration in multiple diseases.

### 2.6. Construction of “Component-Target-Pathway” Network

The intersecting targets and their corresponding components and pathways were imported into Cytoscape 3.9.0 to construct a “component-target-pathway” network. The topological parameters in the network were calculated using the “Network analyzer”, with the importance of a node reflected by its degree value; the more significant the degree value, the more critical the node. The more critical nodes may be key components of valerian volatile oil in treating insomnia. The results show that the perillyl acetate (C38, degree = 42), bis[(6,6-dimethyl-3-bicyclo[3.1.1]hept-2-enyl)methyl] (E)-but-2-enedioate (C37, degree = 31), 2-hydroxy-4a,5-dimethyl-3-prop-1-en-2-yl-2,3,4,5,6,8a-hexahydronaphthalen-1-one (C24, degree = 26), (5beta,7beta,10beta)-3,11-eudesmadien-2-one (C27, degree = 24), ylangenal (C16, degree = 20), 2-tert-butyl-4-ethylphenol (C6, degree = 20), myrtenyl isovalerate (C11, degree = 19), and khusilal (C26, degree = 19) are potential key components of VVO regarding its sedative–hypnotic effects. The “component-target-pathway” network is shown in Figure 3C. More details about the 38 active ingredients are provided in Table S1.

### 2.7. Molecular Docking Results

**Figure 4:**
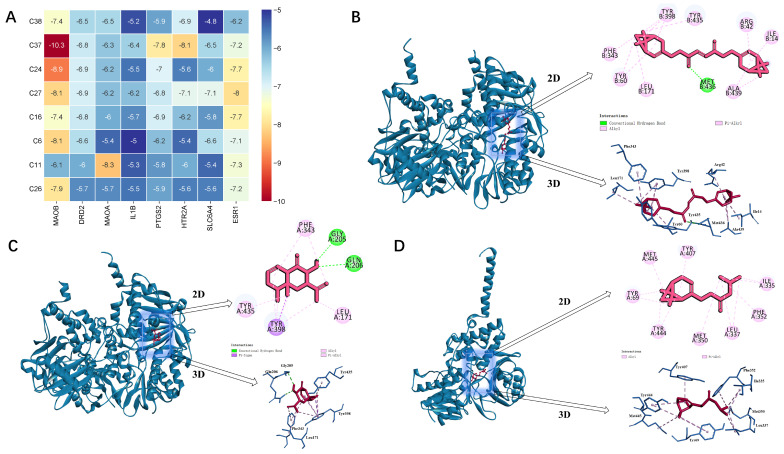
(**A**) Thermogram of molecular docking binding energy. (**B**–**D**) Molecular docking diagrams.

We used AutoDock 1.5.6 software to dock the eight active ingredients with the highest degree values against the eight core proteins within the PPI network. The binding energy obtained by docking the active ingredients to the core targets using the Bioinformatics (https://www.bioinformatics.com.cn/, accessed on 20 March 2024) platform was mapped on a heat map, as shown in Figure 4A. The more stable the binding between the ligand and the receptor, the lower the binding energy of the two. The binding scores for the eight active ingredients of VVO are shown in Table 2.

Overall, the average binding energy of the ligand to the receptor was −6.6 kcal/mol < −5 kcal/mol, and the active ingredients had an excellent ability to bind to the core targets. Among them, MAOB and ESR1 showed a good binding ability to the active ingredients, while the binding ability of the remaining targets to the ingredients varied to some extent. The results show that the binding energy of MAOB-C37 was −10.3 kcal/mol; there were pi-alkyl hydrophobic forces and a conventional hydrogen bond with MET-436. The binding energy of MAOB-C24 was −8.9 kcal/mol; there were pi-alkyl hydrophobic forces, a pi-sigma hydrophobic force with TYR 398, and conventional hydrogen bonds with GLY 205 and GLN 206. The binding energy of MAOA-C11 was −8.3 kcal/mol; the main force was the pi-alkyl hydrophobic force. Their molecular docking models are shown in Figure 4B–D.

### 2.8. Molecular Simulation Results

**Figure 5:**
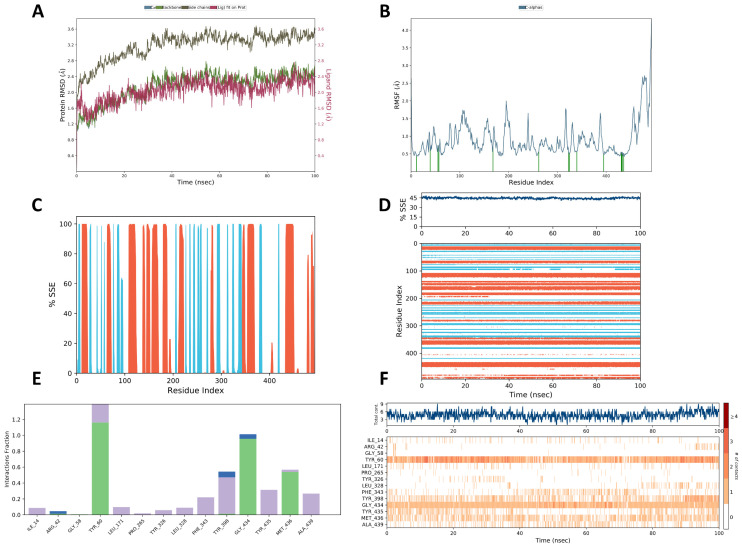
Molecular simulation depicting (**A**) RMSD; (**B**) RMSF. Protein residues that interact with the ligand are marked with green-colored vertical bars; (**C**,**D**) % SSE of MAOB. Orange: alpha-helices, blue: beta-strands; and (**E**,**F**) protein–ligand interactions. Green: H-bonds, purple: hydrophobic, blue: water bridges. Some residues make more than one specific contact with the ligand, which is represented by a darker shade of orange, according to the scale to the right of the plot.

By determining the RMSD of a protein, we can learn about changes in the structural conformation of the protein over time. The RMSD was measured in a simulation of about 200 ns in this study. The average values of RMSD of Cα-MAOB were determined to be around 2.15 Å in the presence of bis[(6,6-dimethyl-3-bicyclo[3.1.1]hept-2-enyl)methyl] (E)-but-2-enedioate, which is within the acceptance range of 1–3 Å (Figure 5A). This suggests that the protein did not undergo a significant conformational change during the simulation. In addition, RMSF values were also plotted against each residue of the MAOB backbone in the presence of bis[(6,6-dimethyl-3-bicyclo[3.1.1]hept-2-enyl)methyl] (E)-but-2-enedioate (Figure 5B). The peaks in the RMSF plot indicate the protein areas that fluctuated the most during the simulation, and the protein residues that interacted with the ligand are marked with green-colored vertical bars. Typically, the tails (N- and C-terminal) fluctuated more than any other part of the protein. This indicates that the stability of the MAOB protein is directly related to the fluctuation of amino acids.

To further investigate, we determined the effect of bis[(6,6-dimethyl-3-bicyclo[3.1.1]hept-2-enyl)methyl] (E)-but-2-enedioate on the secondary structural elements (α-helices and β-strands) of the MAOB protein. The SSE distribution by the residue index throughout the protein structure is shown in Figure 5C. The plot in Figure 5D summarizes the SSE composition in each trajectory frame during the simulation, tracking the assignment of each residue and SSE over time. The helix was 28.66%, the strand was 15.80%, and the total SSE was 44.46%.

Protein–ligand interactions were monitored throughout the simulation. These interactions can be categorized into four types: hydrogen bonds, hydrophobic, ionic, and water bridges. The stacked bar charts (Figure 5E) are normalized over the trajectory process. For example, a value of 0.6 indicates that a specific interaction was maintained for 60% of the simulation time. Values greater than 1.0 are possible, as some protein residue may make multiple contacts with the ligand at the same subtype. The total number of specific interactions between proteins and ligands is shown in the top panel of Figure 5F. The bottom panel shows which residues interacted with the ligand in each trajectory frame. According to the scale at the right of the plot, the darker shade of orange indicates that some residues had many specific contacts with the ligand. It can be seen that more specific interactions were shown by amino acid residues, such as TYR 60, TYR 398, GLY 434, and MET 436.

### 2.9. Effects of VVO on Latency of Sleeping Time and Duration of Sleeping Time in Pentobarbital-Induced Mice

**Figure 6:**
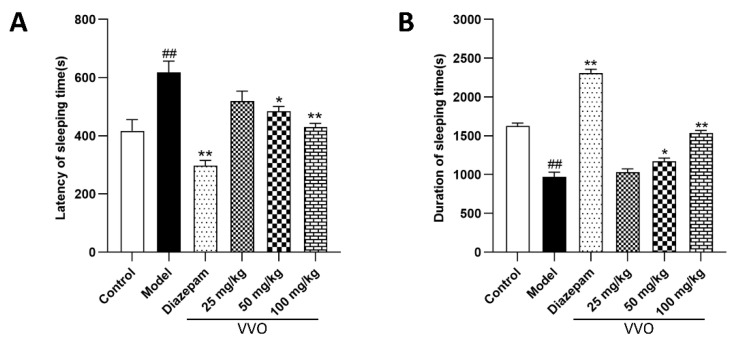
Effects of VVO on pentobarbital-induced latency of sleeping time ((**A**), F(5, 30) = 14.00, *p* < 0.01, *n* = 6) and duration of sleeping time ((**B**), F(5, 30) = 125.2, *p* < 0.01) in mice. Note: Compared with the control group, ## *p* < 0.01; compared with the model group, * *p* < 0.05 and ** *p* < 0.01.

The sleep latency and sleep duration of mice in each group were determined using the classical righting reflex experiment after 6 days of treatment administration. This allowed us to observe whether the sleep of insomniac mice was improved after administering the drug. Compared with the control group (Figure 6), the model group had longer sleep latency (*p* < 0.01) and shorter sleep duration (*p* < 0.01), and the difference between the two groups was significant. The diazepam group had a highly significant shortening of sleep latency (*p* < 0.01) and a highly considerable lengthening of sleep duration (*p* < 0.01) compared with the model group. There was a trend toward reducing sleep latency and slightly increasing sleep duration in the low-dose (25 mg/kg) groups, but no significant differences were found. Sleep latency was significantly shorter (*p* < 0.05, *p* < 0.01), and sleep duration was significantly increased (*p* < 0.05, *p* < 0.01) in the middle (50 mg/kg) and high-dose (100 mg/kg) groups. The above results show that the hypnotic effect of VVO was more significant in the high-dose group, and the effect was not significant in the low-dose groups.

### 2.10. Effect of VVO on Autonomous Activity in Mice

**Figure 7:**
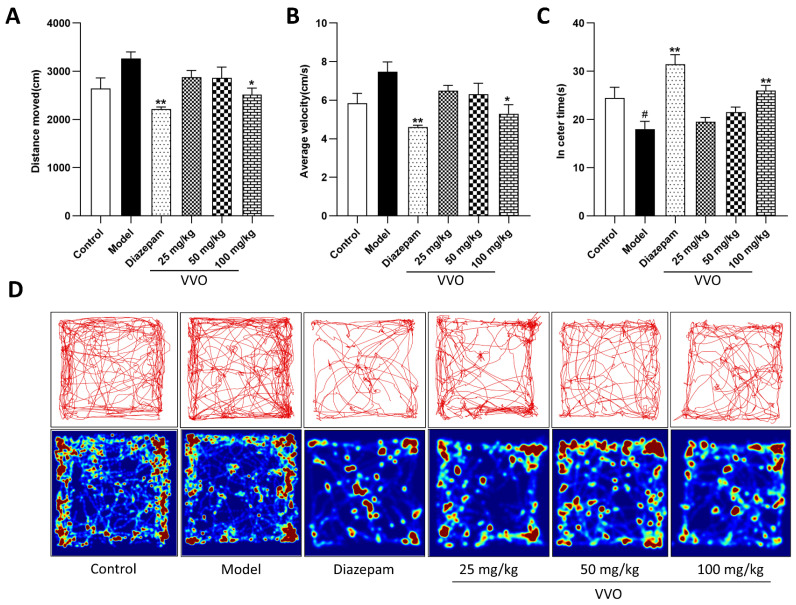
Effects of VVO on distance moved ((**A**), F(5, 53) = 4.902, *p* < 0.01), average velocity ((**B**), F(5, 53) = 5.224, *p* < 0.01, *n* = 10), and in-center time ((**C**), F(5, 53) = 10.16, *p* < 0.01) of mice. Note: Compared with the control group, # *p* < 0.05; compared with the model group, * *p* < 0.05 and ** *p* < 0.01. (**D**) Thermography and trajectories of activity in the open-field experiment for each group of mice.

After the last administration of the drug, open-field tests were performed on groups of mice. The total distance moved, the average speed, and the time spent in the central area were observed to determine the levels of anxiety, autonomy, and exploratory behavior of the mice in the new environment. Compared with the model group (Figure 7A–C), the diazepam group showed highly significant decreases in distance moved and average speed (*p* < 0.01, *p* < 0.01) and considerably higher prolongation of the in-center time (*p* < 0.01). Although there was a decrease in the distance moved and average speed in the low- (25 mg/kg) and medium-dose (50 mg/kg) groups, the difference was not significant, nor was there a significant prolongation in the residence time in the central area. The high-dose (100 mg/kg) groups showed a substantial decrease in the distance moved and mean speed of movement (*p* < 0.05, *p* < 0.05) and a considerable increase in residence time in the central zone (*p* < 0.01). The trajectory and thermogram of mice activity in the open-field experiment are shown in Figure 7D. The mice in the model group had activity trajectories in all regions and were frequently active in the marginal areas. After the drug treatment, the activity trajectories of the low-dose and medium-dose groups did not differ significantly from those of the model group. The activity trajectories of the high-dose and diazepam groups were thinner than those of the model group, and the trajectories in the central region were significantly increased. This indicates that the high-dose groups could alleviate their levels of tension and anxiety and reduce the autonomous activity of insomniac mice.

### 2.11. Effects of VVO on the Level of MAOB Protein in the Cerebral Cortex

**Figure 8:**
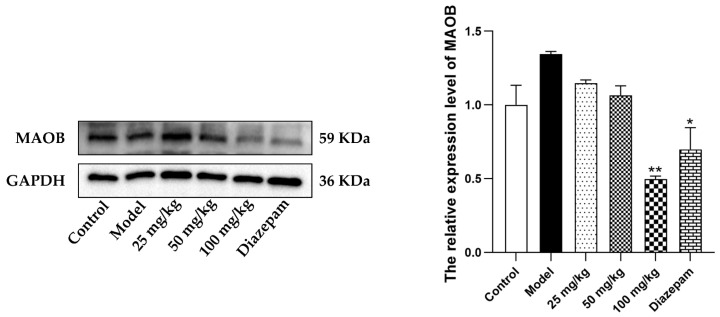
Effects of VVO on the level of MAOB protein in the cerebral cortex (*n* = 3). Note: Compared with the model group, * *p* < 0.05 and ** *p* < 0.01.

From the perspective of the level of MAOB protein in the cerebral cortex (Figure 8), compared with the model group, the level of MAOB protein in the high-dose (100 mg/kg) group was extremely significantly reduced (*p* < 0.01). The level of MAOB protein in the diazepam group was significantly reduced (*p* < 0.05). This indicates that VVO can indeed interact with MAOB and reduce its expression level in the cerebral cortex.

## 3. Discussion

In this study, based on network pharmacology, we found that VVO principally regulated insomnia through the perillyl acetate, bis[(6,6-dimethyl-3-bicyclo[3.1.1]hept-2-enyl)methyl] (E)-but-2-enedioate, 2-hydroxy-4a,5-dimethyl-3-prop-1-en-2-yl-2,3,4,5,6,8a-hexahydronaphthalen-1-one, as well as other components. As a natural substance, valerian has been shown in several studies to have sedative and hypnotic properties in its volatile oil, alcoholic extracts, and aqueous extracts. However, the sedative–hypnotic effects of valerian mono-components have rarely been reported and require further study.

The results from the PPI analysis show that the targets of VVO for the treatment of insomnia mainly involve MAOB, DRD2, MAOA, IL1B, PTGS2, HTR2A, SLC6A4, and ESR1. MAOB and MAOA are two isoforms of monoamine oxidase (MAO) that catalyze the degradation of monoamine neurotransmitters, such as 5-hydroxytryptamine (5-HT), dopamine (DA), and norepinephrine (NE) in the central nervous system (CNS). Although MAOB and MAOA are similar in primary sequence, they have unique substrate and inhibitor specificities. MAOA has a high affinity for 5-HT and NE, whereas MAOB prefers β-phenylethylamine (PEA) as a substrate [30,31]. This difference implies that the two MAO isoenzymes play different roles in brain activity. However, in the absence of MAOA, the substrate specificities of the two MAO isoenzymes overlap, allowing MAOB to also metabolize 5-HT and other monoamines [32]. It was found that the knockout of the MAOB gene was able to reduce anxiety-like responses in mice through regional elevation of PEA [33]. VVO acts as a sedative–hypnotic treatment by modulating MAOA, preventing 5-HT from becoming the metabolite 5-hydroxyindoleacetic acid (5-HIAA). The 5-HT2 receptors are neurotransmitter receptors that are closely associated with a variety of physiological processes, including mood regulation, cognitive function, and pain perception. Multiple subtypes of 5-HT2 receptors are found in different regions of the CNS [34]. HTR2A, one of the isoforms, belongs to the G protein-coupled receptors and can mediate the excitatory effects of 5-HT. It has been in the spotlight as a significant target for atypical antipsychotics [35]. Research has shown that stress can impair the function of 5-HT2A receptors, which affects the release of GABA and then leads to emotional and behavioral problems [36]. SLC6A4 is a 5-HT transporter protein that transports the 5-HT from the synaptic gap to the presynaptic neuron and plays a crucial role in 5-HT homeostasis in the CNS. Genetic variants in the SLC6A4 gene exist in different individuals, and these variants are associated with an increased risk of posttraumatic stress disorder, depression, and obsessive–compulsive disorder [37,38].

GO enrichment analysis showed that VVO modulates insomnia mainly through biological processes, such as trans-synaptic signaling, synaptic signaling, anterograde trans-synaptic signaling, and chemical synaptic transmission. KEGG pathway enrichment analysis showed that the major pathways for treating insomnia were the neuroactive ligand–receptor interaction, serotonergic synapse, calcium signaling pathway, and nicotine addiction pathway. It was found that rosa damascena essential oil could reverse neuronal damage in the hippocampus of mice through the serotonergic synapse signaling pathway, restoring neural tissues and protecting cognitive functions, thus exerting antidepressant effects [39]. The caryophyllene in valerian essential oil can upregulate the 5-HT1AR receptor, allowing more 5-HT to couple with the G protein-coupled receptor, thereby modulating the downstream pathway and increasing the expression of 5-HT and GABA, which in turn improves insomnia [40]. This led to the discovery that serotonergic synaptic pathways play an important role in neurological disorders.

The molecular docking results show that the average binding energies of the eight active ingredients to the eight core proteins were <−6.6 kcal/mol, indicating strong binding between the active ingredients and the core targets. In addition, molecular dynamics simulations were performed for the complex of MAOB-C37(bis[(6,6-dimethyl-3-bicyclo[3.1.1]hept-2-enyl)methyl] (E)-but-2-enedioate), which has the lowest binding energy. The results show that MAOB forms a stable binding with bis[(6,6-dimethyl-3-bicyclo[3.1.1]hept-2-enyl)methyl] (E)-but-2-enedioate. The experimental results of Western blotting indicated that the level of MAOB protein in the high-dose (100 mg/kg) group was significantly reduced. This further confirms that VVO can indeed interact with MAOB and decrease its expression level in the cerebral cortex.

In general, standard models of insomnia include the use of physical and pharmacological methods of sleep deprivation. The PCPA insomnia mouse model was used in this study. PCPA is a 5-HT synthesis inhibitor that inhibits 5-HT synthesis in the brain, which in turn causes insomnia. The open-field test is a classical method used to evaluate autonomous behavior in mice. In general, reduced autonomous activity in mice indicates a soothing effect of the drug. Most sleep experiments are based on the synergistic effects of sedative medications and barbiturates. Sodium pentobarbital is a CNS depressant with sedative–hypnotic properties. This study used the open-field test and sodium pentobarbital synergistic sleep test to evaluate autonomous behavior, sleep latency, and sleep duration in mice. The results show that gavage administration of high doses of VVO can significantly shorten the latency of sleep time and prolong the duration in mice. The total distance moved by the mice in the open-field test was reduced, the average speed was lowered, and the residence time in the central area became longer. The sedative–hypnotic effects of low and medium doses of VVO were not significant.

In this study, we integrated network pharmacology, molecular docking, molecular dynamics simulation, and pharmacodynamics experiments to confirm the sedative–hypnotic effects of VVO. The results reveal that VVO synergistically exerts therapeutic effects on insomnia through multiple components, multiple targets, and multiple pathways. We could predict the role of VVO in modulating the serotonergic synaptic pathway in the treatment of insomnia, and this modulation was associated with MAOB, MAOA, PTGS2, HTR2A, and SLC6A4. However, there are some limitations to this experiment, as it only assessed the pharmacodynamics in mice, and more in vivo and in vitro studies are needed to further elucidate the function and mechanisms of action of VVO.

## 4. Materials and Methods

### 4.1. Main Drugs and Reagents

Valerian root (Guizhou Miaoyao Biotech; Guizhou, China), diazepam injection (Tianjin Jinyao Pharmaceutical, batch number: H12020957; Tianjin, China), 4-Chloro-DL-phenylalanine (Shanghai Acmec Biochemical Technology, batch number: C435386BE; Shanghai, China), sodium pentobarbital (Merck, batch number: H82-F158; Darmstadt, Germany), Tween 80 (Shanghai Acmec Biochemical Technology, batch number: T54535F5B; Shanghai, China), 0.9% saline (Shandong Qidu Pharmaceutical, batch number: R9B23082102; Zibo, China), anhydrous sodium sulfate (Guangzhou Chemical Reagent Factory, batch number: 20140901-1; Guangzhou, China), TBS buffer (BOSTER, batch number: 19J14B31; Shanghai, China), BCA protein quantification kit (Abbkine, batch number:ATXH27161; Wuhan, China), protein prestained (Thermo, batch number:2969018; Billerica, MA, USA), PVDF membrane (Millipore, batch number: 0000333516; Billerica, MA, USA), skimmed milk powder (Biosharp, batch number: 24208847; Beijing, China), femto-sensitive ECL solution (MIKX, batch number: 20240515; Shenzhen, China), MAOB antibody (Affinity, batch number: 7K35705; Melbourne, Australia), GAPDH antibody (HUABIO, batch number: H661060013; Hangzhou, China), and goat anti-rabbit HRP-labeled secondary antibody (Sinobiological, batch number: HO17AP2401; Beijing, China) were the materials used for the experiments.

### 4.2. Animals

A total of 60 Kunming mice aged 5–6 weeks (SPF grade, 30 male and 30 female, 25~30 g) were used in the experimental study. The animals were purchased from Guangdong Weitong Lihua Laboratory Animal Technology (license number: SCXK(YUE)2022-0063; Foshan, China). The animals were exposed to light for 12 h daily with alternating black and white light cycles. The experiments were started after 3 days of adaptive feeding. The experiment received approval from the ethics committee of Guangzhou Medical University (approval number: GY2023-634).

### 4.3. Extraction of Valerian Volatile Oil

First, 280 g of dried valerian roots and rhizomes were crushed into powder. Then, they were filtered through a 24-mesh screen and subjected to supercritical CO_2_ extraction (SFE-2, Applied Separations; Allentown, PA, USA). The extraction parameters were as follows: extraction pressure of 21 MPa, extraction temperature of 45 °C, extraction time of 2 h, and CO_2_ flow rate of 1.5–2.0 L/min.

### 4.4. GC-MS Analysis

The supercritical extract was analyzed and identified using GC-MS (8890-5977B, Agilent; Santa Clara, CA, USA). The chromatographic parameters were as follows: HP-5 MS UI (30 m × 0.25 mm × 0.25 μm) with a programmed ramp-up mode starting at 60 °C and increasing to 110 °C at 2 °C/min, then to 140 °C at 1 °C/min, and ultimately reaching 250 °C at 2 °C/min. The shunt ratio was set to 10:1, with helium as the carrier gas at a flow rate of 1 mL/min, an injection volume of 1 µL, and an inlet temperature of 250 °C. The mass spectrometry conditions were as follows: transmission line temperature of 280 °C; ion source temperature of 230 °C; solvent delay time of 3 min; scan mass range of 20~450 amu.

### 4.5. Screening of Active Ingredients and Target Genes for VVO

Based on the GC-MS results, the SwissADME (http://www.swissadme.ch/, accessed on 1 March 2024) platform was used to screen active compounds with better oral bioavailability and drug-like properties. The screening criteria were “High” for GI absorption under pharmacokinetics and “Yes” for two or more of the five drug predictions (Lipinski, Ghose, Veber, Egan, Muegge) under drug-likeness [41]. The potential targets of the compounds were predicted in the SwissTargetPrediction (http://www.swisstargetprediction.ch/, accessed on 1 March 2024) database using probability > 0 as a screening condition.

### 4.6. Screening for Insomnia Targets and Common Targets

Using the GeneCards (https://www.genecards.org/, accessed on 5 March 2024), DrugBank (https://go.drugbank.com/, accessed on 5 March 2024), and TTD (https://db.idrblab.net/ttd/, accessed on 5 March 2024) databases, we screened targets related to insomnia. “Insomnia” was entered as the search keyword. The gene targets retrieved were a result of the three merged disease databases, and all duplicate values were deleted. After the disease target was obtained, the BioLadder (https://www.bioladder.cn/, accessed on 5 March 2024) platform was used to identify the common targets between the disease and the components, and a Venn diagram was created.

### 4.7. PPI Network Construction and Core Target Screening

The intersection targets were imported into the STRING (https://cn.string-db.org/, accessed on 10 March 2024) database to construct a PPI network model using biological species set to “Homo sapiens”. The confidence was >0.4, and unconnected nodes were hidden. From this, a protein–protein interaction network (PPI) was obtained, and the TSV file format was downloaded to construct a PPI network using Cytoscape 3.9.0 software. Then, the CytoNCA plug-in was used to perform topological analysis and screen out the core targets.

### 4.8. GO and KEGG Pathway Enrichment Analysis

The Metascape (https://metascape.org/, accessed on 15 March 2024) database was used to analyze the typical targets of VVO and insomnia for GO functional and KEGG pathway enrichment analysis. GO enrichment analysis consists of three modules: biological process (BP), cell composition (CC), and molecular function (MF). Cytoscape software was used to construct a “component-target-pathway” network with the top 20 pathways, their overlapping targets, and the corresponding active substances.

### 4.9. Molecular Docking

Utilizing the screened core targets and critical ingredients, the vital active ingredients were used as small molecule ligands, and the core targets were used as prominent molecule receptors. Molecular docking was carried out using AutoDock Vina 1.1.2, and the docking results were visualized in docking models using Discovery Studio 19.1.0 software.

### 4.10. Molecular Dynamics Simulation

The Desmond program selected the docking complexes with the best binding ability among the molecular docking results for molecular dynamics simulations (Schrodinger, LLC; New York, NY, USA). Na+ and Cl^−^ ions were added to each system to neutralize the charges. To minimize and relax the system, the NPT (number of atoms (N), pressure (P), and temperature (T) set) from the Desmond program was used. When retraining the complex, the system was first equilibrated for 100 ns using the NVT ensemble. Using an NPT ensemble, a short run for equilibration and minimization was performed for 20 ns. In this experiment, the stability of the molecular dynamics simulations was calculated using various parameters, such as root mean square deviation (RMSD), root mean square fluctuation (RMSF), and number of hydrogen bonds.

### 4.11. Establishing Mice Models of Insomnia and Treatment

A total of 60 KM mice, after acclimatization feeding, were randomly divided into a regular group, a model group, a diazepam group, and a valerian volatile oil group (with a low-dose group, medium-dose group, and high-dose group) with 10 mice in each group. Except for the standard group, all mice were injected intraperitoneally at the same time every morning with a saline solution of PCPA at 300 mg/kg for 2 consecutive days to establish the insomnia model in mice. Compared with the normal group, the mice exhibiting phenomena such as dull hair, agitation, constant activity, and the disappearance of diurnal rhythm indicated successful modeling. After the establishment of the PCPA insomnia model, the valerian low-, medium-, and high-dose groups and the diazepam group were treated by gavage at doses of 25 mg/kg, 50 mg/kg, 100 mg/kg, and 2 mg/kg, respectively, and the standard and model groups were treated by gavage with equal volumes of distilled water. The administration volume was 0.1 mL/10 g. The drug was administered by gavage once a day for 7 consecutive days.

### 4.12. Pentobarbital-Induced Sleeping Test

The mice in each group were injected intraperitoneally with pentobarbital sodium at a dose of 45 mg/kg 30 min after the 6th gavage administration. The mice were placed belly up, and the time it took for the disappearance and recovery of the flip reflex was recorded. The sleep criterion was the disappearance of the flip reflex for more than 1 min, and recovery of the flip reflex was indicated by the ability of the mouse to turn its body more than 3 times within 1 min, which was taken as the awakening criterion. Sleep latency and sleep duration were calculated for each group of mice.

### 4.13. Open-Field Test

After 30 min following the final drug delivery, the open-field test was conducted on each group of mice. The mice were acclimatized to the environment for 3 min. Their behavioral metrics, including the total distance traveled, average speed, and duration of stay in the center area, were recorded during a 5-min interval, and the intergroup differences among the mice were analyzed.

### 4.14. Western Blot

The cerebral cortex was separated, and total protein was extracted. Tissue protein concentration was determined by the BCA method, and the MAOB protein was separated by 10% SDS-PAGE gel electrophoresis. After a 100 min electrotransfer to a PVDF membrane, the membrane was removed and rinsed in TBS buffer (TBS-T) for 10 min, then blocked with blocking solution for 1 h (TBS-T buffer containing 5% skimmed milk powder). After incubation at room temperature, the blocking solution was discarded, and primary antibodies MAOB (1:1000) were added. After incubation at 4 °C overnight, TBS-T was washed 3 times for 10 min each. Then, a secondary antibody (1:5000) was added and incubated for 2 h, washed in TBS-T 3 times for 10 min each time, and finally, ECL was added for color development solution.

### 4.15. Statistical Analysis

The test results were expressed as the means ± SEMs. Graph Pad Prism 8.0 software was used for graphing. One-way ANOVA was used for significance tests among multiple groups, and a *t*-test was used for comparing the sample means between two groups. *p* < 0.05 was taken as the level of significance.

## 5. Conclusions

In summary, our research findings have elucidated that VVO treats insomnia through a variety of components and multiple targets, with MAOB, MAOA, SLC6A4, HTR2A, and others at serotonergic synapses playing a vital role in the process of VVO treating insomnia. These research results provide theoretical support and research data for the clinical application of VVO in treating insomnia, and preliminary reveal the pharmacodynamic basis and pharmacological mechanism of action of VVO in insomnia. However, our study also has some limitations. Network pharmacological studies are generally based on computational predictions, but in future research, it will be necessary to integrate other more direct and precise methods (such as high-throughput sequencing) to validate our findings.

## Figures and Tables

**Table 1:** Valerian volatile oil active compounds.

Number	Name	Retention Time (min)	Chemical Formula	Molecular Mass (g/mol)
C1	2,3-Butanediol	3.289	C_4_H_10_O_2_	90.12
C2	(+)-Borneol	16.71	C_10_H_18_O	154.25
C3	(-)-Myrtenol	18.55	C_10_H_16_O	152.23
C4	Bornyl acetate	23.907	C_12_H_20_O_2_	196.29
C5	2-formyl-3-methyl-alpha-methylene-Cyclopentaneacetaldehyde	24.676	C_10_H_14_O_2_	166.22
C6	2-tert-Butyl-4-ethylphenol	38.512	C_12_H_18_O	178.27
C7	beta-Ionone	38.662	C_13_H_20_O	192.3
C8	3′,4′-Dimethoxyacetophenone	39.216	C_10_H_12_O_3_	180.2
C9	Bornyval	41.294	C_15_H_26_O_2_	238.37
C10	Shyobunol	41.809	C_15_H_26_O	222.37
C11	Myrtenyl isovalerate	45.213	C_15_H_24_O_2_	236.35
C12	Spathulenol	46.493	C_15_H_24_O	220.35
C13	Isospathulenol	51.483	C_15_H_24_O	220.35
C14	Ent-Spathulenol	52.216	C_15_H_24_O	220.35
C15	1,1,4,7-Tetramethyldecahydro-1H-cyclopropa[e]azulene-4,7-diol	60.236	C_15_H_26_O_2_	238.37
C16	Ylangenal	62.147	C_15_H_22_O	218.33
C17	1-methyl-4-prop-1-en-2-ylspiro[4.5]dec-8-ene-8-carbaldehyde	63.426	C_15_H_22_O	218.33
C18	2,2,4-trimethylcyclohex-3-ene-1-carbaldehyde	66.888	C_10_H_16_O	152.23
C19	.beta.-Santalol	67.473	C_15_H_24_O	220.35
C20	dehydro-Cyclolongifolene oxide	68.083	C_15_H_22_O	218.33
C21	alpha-Costol	68.693	C_15_H_24_O	220.35
C22	Dihydrocarveol	70.22	C_10_H_18_O	154.25
C23	Humulenol II	70.852	C_15_H_24_O	220.35
C24	2-hydroxy-4a,5-dimethyl-3-prop-1-en-2-yl-2,3,4,5,6,8a-hexahydronaphthalen-1-one	73.218	C_15_H_22_O_2_	234.33
C25	1-O-[(6,6-dimethyl-2-bicyclo[3.1.1]hept-2-enyl)methyl] 5-O-(3-methylbutan-2-yl) pentanedioate	74.864	C_20_H_32_O_4_	336.5
C26	Khusilal	75.302	C_14_H_18_O	202.29
C27	(5beta,7beta,10beta)-3,11-Eudesmadien-2-one	76.066	C_15_H_22_O	218.33
C28	alpha-Valerenol	76.344	C_15_H_24_O	220.35
C29	4-Isopropenyl-4,7-dimethyl-1-oxaspiro[2.5]octane	77.512	C_12_H_20_O	180.29
C30	14-Hydroxy-9-epi-(E)-caryophyllene	83.282	C_15_H_24_O	220.35
C31	1-Ethoxy-2-chloro-2-2-(thienyl)cyclopropane	84.19	C_9_H_11_ClOS	202.7
C32	4-(3-methyl-1H-pyrazol-1-yl)aniline	85.416	C_10_H_11_N_3_	173.21
C33	beta-Costol	91.547	C_15_H_24_O	220.35
C34	6-(3-hydroxyprop-1-en-2-yl)-4,8a-dimethyl-2,3,5,6,7,8-hexahydro-1H-naphthalen-2-ol	94.38	C_15_H_24_O_2_	236.35
C35	N-[2-(4-Chloro-2-nitro-phenylamino)-ethyl]-benzenesulfonamide	100.372	C_14_H_14_ClN_3_O_4_S	355.8
C36	2,6,6-trimethylbicyclo[3.1.1]hept-3-en-2-ol	111.513	C_10_H_16_O	152.23
C37	bis[(6,6-dimethyl-3-bicyclo[3.1.1]hept-2-enyl)methyl] (E)-but-2-enedioate	117.801	C_24_H_32_O_4_	384.5
C38	Perillyl acetate	118.172	C_12_H_18_O_2_	194.27

**Table 2:** The binding scores for the eight active ingredients of VVO.

Ingredients	Proteins	PDB Number	Binding Scores (kcal/mol)
Perillyl acetate (C38)	MAOB	2xfu	−7.4
	DRD2	6cm4	−6.5
	MAOA	2z5y	−6.5
	IL1B	1hib	−5.2
	PTGS2	5f19	−5.9
	HTR2A	7wc4	−6.9
	SLC6A4	6dzv	−4.8
	ESR1	2bj4	−6.2
bis[(6,6-dimethyl-3-bicyclo[3.1.1]hept-2-enyl)methyl] (E)-but-2-enedioate (C37)	MAOB	2xfu	−10.3
	DRD2	6cm4	−6.8
	MAOA	2z5y	−6.3
	IL1B	1hib	−6.4
	PTGS2	5f19	−7.8
	HTR2A	7wc4	−8.1
	SLC6A4	6dzv	−6.5
	ESR1	2bj4	−7.2
2-hydroxy-4a,5-dimethyl-3-prop-1-en-2-yl-2,3,4,5,6,8a-hexahydronaphthalen-1-one (C24)	MAOB	2xfu	−8.9
	DRD2	6cm4	−6.9
	MAOA	2z5y	−6.2
	IL1B	1hib	−5.5
	PTGS2	5f19	−7.0
	HTR2A	7wc4	−5.6
	SLC6A4	6dzv	−6.0
	ESR1	2bj4	−7.7
(5beta,7beta,10beta)-3,11-Eudesmadien-2-one (C27)	MAOB	2xfu	−8.1
	DRD2	6cm4	−6.9
	MAOA	2z5y	−6.2
	IL1B	1hib	−6.2
	PTGS2	5f19	−6.8
	HTR2A	7wc4	−7.1
	SLC6A4	6dzv	−7.1
	ESR1	2bj4	−8.0
Ylangenal (C16)	MAOB	2xfu	−7.4
	DRD2	6cm4	−6.8
	MAOA	2z5y	−6.0
	IL1B	1hib	−5.7
	PTGS2	5f19	−6.9
	HTR2A	7wc4	−6.2
	SLC6A4	6dzv	−5.8
	ESR1	2bj4	−7.7
2-tert-Butyl-4-ethylphenol (C6)	MAOB	2xfu	−8.1
	DRD2	6cm4	−6.6
	MAOA	2z5y	−5.4
	IL1B	1hib	−5.0
	PTGS2	5f19	−6.2
	HTR2A	7wc4	−5.4
	SLC6A4	6dzv	−6.6
	ESR1	2bj4	−7.1
Myrtenyl isovalerate (C11)	MAOB	2xfu	−6.1
	DRD2	6cm4	−6.0
	MAOA	2z5y	−8.3
	IL1B	1hib	−5.3
	PTGS2	5f19	−5.8
	HTR2A	7wc4	−6.0
	SLC6A4	6dzv	−5.4
	ESR1	2bj4	−7.3
Khusilal (C26)	MAOB	2xfu	−7.9
	DRD2	6cm4	−5.7
	MAOA	2z5y	−5.7
	IL1B	1hib	−5.5
	PTGS2	5f19	−5.9
	HTR2A	7wc4	−5.6
	SLC6A4	6dzv	−5.6
	ESR1	2bj4	−7.2

## Data Availability

The data that support the findings of this study are available from the corresponding author upon reasonable request.

## Supplementary Materials

The following supporting information can be downloaded at: https://www.mdpi.com/article/10.3390/ijms26041726/s1.

## Abbreviations

BP	Biological process
CC	Cell composition
CNS	Central nervous system
DA	Dopamine
DRD2	Dopamine receptor d2
ESR1	Estrogen receptor 1
GABA	Gamma-aminobutyric acid
GC-MS	Gas chromatography–mass spectrometry
GLU	Glutamic acid
GI	Gastrointestinal
GO	Gene ontology
HTR2A	5-Hydroxytryptamine receptor 2a
IL1B	Interleukin 1 beta
KEGG	Kyoto Encyclopedia of Genes and Genomes
MAOA	Monoamine oxidase a
MAOB	Monoamine oxidase b
MF	Molecular function
NE	Norepinephrine
PCPA	p-Chlorophenylalanine
PEA	β-Phenylethylamine
PPI	Protein–protein interaction
PTGS2	Prostaglandin-endoperoxide synthase 2
RMSD	Root mean square deviation
RMSF	Root mean square fluctuation
SLC6A4	Solute carrier family 6 member 4
SSE	Secondary structure elements
TTD	Therapeutic target database
VVO	Valerian volatile oil
