# Supplementary material for: Exploring the Effects and Mechanisms of Valerian Volatile Oil in Treating Insomnia Using Network Pharmacology, Molecular Docking, and Molecular Dynamics Simulation-Based Approaches
Source: Int J Mol Sci. 2025 Feb 18;26(4):1726. doi: 10.3390/ijms26041726 (PMC11855732; doi:10.3390/ijms26041726)
Supplement: Supplementary file 1 [file ijms-26-01726-s001.zip › ijms-3430774-supplementary.pdf]

Table S1. Relevant information about 38 active ingredients in the "component-target-pathway".

Number	Name	Degree	BetweennessCentrality	ClosenessCentrality
1	C38	42	0.126752165	0.450424929
2	C37	31	0.056619449	0.415143603
3	C24	26	0.067245321	0.419525066
4	C27	24	0.043950027	0.406649616
5	C16	20	0.026472825	0.402531646
6	C6	20	0.050293661	0.390663391
7	C11	19	0.040398206	0.400503778
8	C26	19	0.026694072	0.396508728
9	C18	17	0.017823364	0.394540943
10	C33	17	0.024253965	0.38313253
11	C35	16	0.040364553	0.400503778
12	C9	15	0.035621922	0.388753056
13	C2	14	0.027004153	0.384987893
14	C30	14	0.014385649	0.37236534
15	C36	13	0.008959547	0.37236534
16	C21	12	0.007416078	0.370629371
17	C5	12	0.012984751	0.377672209
18	C15	11	0.00932228	0.374117647
19	C17	11	0.007776423	0.37236534
20	C13	10	0.005435048	0.367205543
21	C22	10	0.005762179	0.374117647
22	C8	10	0.033557824	0.375886525
23	C19	9	0.004360639	0.365517241
24	C31	9	0.011418162	0.375886525
25	C34	9	0.003292173	0.358916479
26	C7	8	0.031023783	0.355704698
27	C10	7	0.01505046	0.357303371
28	C23	7	0.004773672	0.355704698
29	C25	7	0.00401778	0.370629371
30	C3	7	0.002004371	0.362186788
31	C28	5	0.001466605	0.350993377
32	C12	4	0.001419659	0.33901919
33	C4	4	0.003718633	0.344902386
34	C14	3	0.00039677	0.323828921
35	C20	1	0	0.326488706
36	C29	1	0	0.261943987
37	C32	1	0	0.203065134

Note: Arrange according to degree value from largest to smallest. C1 does not participate in the interaction, therefore it is excluded from this table.
